# Early Detection of 5 Neurodevelopmental Disorders of Children and Prevention of Postnatal Depression With a Mobile Health App: Observational Cross-Sectional Study

**DOI:** 10.2196/58565

**Published:** 2024-06-18

**Authors:** Fabrice Denis, Florian Le Goff, Madhu Desbois, Agnes Gepner, Guillaume Feliciano, Denise Silber, Jean-David Zeitoun, Guedalia Peretz Assuied

**Affiliations:** 1 Institute for Smarthealth INeS Le Mans France; 2 Kelindi Lille France; 3 Basil Strategies Paris France; 4 Centre d’Epidémiologie Clinique Hôtel Dieu Hospital Assistance Publique Hopitaux de Paris Paris France; 5 Service de Pédopsychiatrie Centre Hospitalier des Pyrénées Pau France

**Keywords:** mobile phone, pediatric, infant, baby, neonate, newborn, toddler, child, early detection, app, application, screening, algorithm, NDD, neurodevelopmental disorder, autism, ASD, autism spectrum disorder, attention deficit/hyperactivity disorder, ADHD, attention deficit, PND, postnatal depression, mHealth, mobile health, real-world study, smartphone, dyspraxia, delayed, language, dyslexia, incidence, prevalence

## Abstract

**Background:**

Delay in the diagnosis of neurodevelopmental disorders (NDDs) in toddlers and postnatal depression (PND) is a major public health issue. In both cases, early intervention is crucial but too rarely implemented in practice.

**Objective:**

Our goal was to determine if a dedicated mobile app can improve screening of 5 NDDs (autism spectrum disorder [ASD], language delay, dyspraxia, dyslexia, and attention-deficit/hyperactivity disorder [ADHD]) and reduce PND incidence.

**Methods:**

We performed an observational, cross-sectional, data-based study in a population of young parents in France with at least 1 child aged <10 years at the time of inclusion and regularly using Malo, an “all-in-one” multidomain digital health record electronic patient-reported outcome (PRO) app for smartphones. We included the first 50,000 users matching the criteria and agreeing to participate between May 1, 2022, and February 8, 2024. Parents received periodic questionnaires assessing skills in neurodevelopment domains via the app. Mothers accessed a support program to prevent PND and were requested to answer regular PND questionnaires. When any PROs matched predefined criteria, an in-app recommendation was sent to book an appointment with a family physician or pediatrician. The main outcomes were the median age of the infant at the time of notification for possible NDD and the incidence of PND detection after childbirth. One secondary outcome was the relevance of the NDD notification by consultation as assessed by health professionals.

**Results:**

Among 55,618 children median age 4 months (IQR 9), 439 (0.8%) had at least 1 disorder for which consultation was critically necessary. The median ages of notification for probable ASD, language delay, dyspraxia, dyslexia, and ADHD were 32.5 (IQR 12.8), 16 (IQR 13), 36 (IQR 22.5), 80 (IQR 5), and 61 (IQR 15.5) months, respectively. The rate of probable ADHD, ASD, dyslexia, language delay, and dyspraxia in the population of children of the age included between the detection limits of each alert was 1.48%, 0.21%, 1.52%, 0.91%, and 0.37%, respectively. Sensitivity of alert notifications for suspected NDDs as assessed by the physicians was 78.6% and specificity was 98.2%. Among 8243 mothers who completed a PND questionnaire, highly probable PND was detected in 938 (11.4%), corresponding to a reduction of –31% versus our previous study without a support program. Suspected PND was detected a median 96 days (IQR 86) after childbirth. Among 130 users who filled in the satisfaction survey, 99.2% (129/130) found the app easy to use and 70% (91/130) reported that the app improved follow-up of their child. The app was rated 4.8/5 on Apple’s App Store.

**Conclusions:**

Algorithm-based early alerts suggesting NDDs were highly specific with good sensitivity as assessed by real-life practitioners. Early detection of 5 NDDs and PNDs was efficient and led to a possible 31% reduction in PND incidence.

**Trial Registration:**

ClinicalTrials.gov NCT06301087; https://www.clinicaltrials.gov/study/NCT06301087

## Introduction

After birth, the mother-child dyad can be impacted by impairments that remain undetected or are detected too late. Among these impairments, a neurodevelopmental disorder (NDD) such as autism spectrum disorder (ASD) affects 1 in 166 children [1]. The average time to diagnosis is approximately 4 to 6 years, whereas consensus statements indicate that a diagnosis could be made as early as 12 or 18 months of age [2-6]. Interestingly, parents are the main contributors to the NDD screening of their children [7]. Other disorders that deserve early screening are dyspraxia, language delay, dyslexia, and attention-deficit/hyperactivity disorder (ADHD) [8-11]. It is crucial to provide parents with screening tools and to recommend that they consult the physician at the first symptoms to treat them as early as possible.

Postnatal depression (PND) in mothers is another example of an underdiagnosed disorder with severe consequences. PND—an episode of depression occurring during the first year after childbirth—has a prevalence of 17% and may have a negative impact on the synchrony or receptivity loop that is crucial to the proper neurodevelopment of the baby [12,13].

All these disorders can benefit tremendously from early detection by electronic patient-reported outcome questionnaires for parents and their children, which would enable early intervention.

We thus developed Malo, an “all-in-one” multidomain digital health record electronic patient-reported outcome app for smartphones, aiming to facilitate early screening of NDDs in children from birth to the age of 10 years and PND in mothers. We previously reported the results of a first observational, cross-sectional, data-based study in a population of 4242 children in 2022, showing a sensitivity of the alert notifications of suspected NDDs (possible ASD, vision, audition, socialization, language, or motor disorders) as assessed by the physicians of 100%, and a specificity of 73.5%. We also reported an earlier detection of PND in 907 mothers showing an incidence rate of 16.6% [14]. Since these results, changes in algorithms were performed to improve the specificity of NDD screening to avoid false positive results and we added early and continuous advice and support programs after childbirth to mothers to reduce the incidence of PND.

We thus report here the results of the revised algorithm aiming to be more specific for the screening of 5 NDDs (ASD, language delay, dyspraxia, dyslexia, and ADHD) and to assess the impact of the app and support program on the reduction in PND incidence.

## Methods

### Ethical Considerations

We ran an ecological, observational, cross-sectional, data-based study. Our study was approved by the French National Health Data Institute (Health Data Hub approval 16562971), which ensures ethical conduct in human participant research regarding data confidentiality and safety. The approval number for our human participant’s review was F20210420115840. The study complied with good epidemiology practices defined by the Association for French-speaking Epidemiologists. All the users received written information on our primary data collection purpose and the ability to be included in a secondary analysis in a deidentified manner—as specified in the app’s general conditions and privacy policy. However, users did opt in explicitly to be included in this specific study. Data were deidentified and aggregated at the server level before being passed to the researchers.

Data collection was embedded in the app. Data were collected in a French-certified health data cloud, as requested by local laws. Respondents self-entered the age and gender of their infants. The app also allowed for the entry of the children’s height, weight, vaccination status, medical background, and ongoing or previous treatments. No identifying data were used to produce our analysis, with emails, names, and exact dates of birth being segregated in other databases. For instance, the date of birth was converted to the child’s age in days at the time of answer in the data set. No compensation was provided to participants in this study.

### Population

Malo is a mobile health (mHealth) app available on iOS and Android—only available in France at the moment. The kick-off of the first version of the app was historically initiated by a French national media campaign that was disseminated through social media between November 11 and 18, 2021. Details of kick-off modalities are provided by Denis et al [14]. The places of recruitment were multiple—3 maternity units, a dozen daycare centers, 15,000 followers’ social media accounts, and some insurers’ marketing efforts.

For this study, we included 55,618 users matching the criteria and agreeing to participate between May 1, 2022, and February 8, 2024. Enrollment in the study was strictly optional. Recruitment was open with no exclusion criteria. The inclusion criteria were to download the app, to have at least 1 child at least 10 years of age, and to provide informed consent (in-app). We extended the inclusion age of children up to 10 years to allow assessment of the incidence of dyspraxia/dyslexia disorders that can occur during this period.

### Data Collection

Questionnaires and scales, each containing 25-50 questions assessing neurodevelopment skills, were automatically submitted every month from birth to 9 months, then at 11, 12, 16, 18, 21, 24, 30, and 36 months and every 6 months until 10 years of age. Questionnaires were focused on sociability, attention or activity, motricity, language of their infants, and possible ASD to screen for ASD, language delay, dyspraxia, dyslexia, and ADHD.

Questionnaires and notifications were based on French health authorities’ reports, international recommendations, and experts’ agreements [8,15,16]. The questionnaire for the screening of PND was submitted to mothers every 2 to 4 weeks for 7 months after childbirth, using a modified questionnaire of the Edinburgh Postnatal Depression Scale adapted for self-assessment.

### Threshold-Based In-App Notification and Outcome of NDD Module of the App

Notifications were sent automatically to the user if some symptoms matched predefined criteria and a physician consultation was recommended. Regarding NDDs, once a threshold of concern was reached, a notification was sent recommending that the mothers discuss their symptoms with their general practitioner or pediatrician.

The main outcome of the study was the median age of possible NDD notification of infants. The secondary outcomes were, first, user satisfaction regarding app experience and the level of support in child follow-up and second, relevance of the NDD notifications assessed by physicians, using a specific optional survey asking parents the following questions: (1) “In the past month, did your doctor detect a developmental disorder in your child during a follow-up consultation? YES or NO”; (2) “If you had a notification from Malo, did you follow the recommendation of the app to visit a physician? YES or NO”; and (3) “Which of the following reflects the physicians’ reply? (A) The notification is not relevant, (B) the notification is relevant and a medical surveillance of the evolution of the symptom is needed, (C) the advice of an expert is needed, or (D) a treatment is indicated.”

### Threshold-Based In-App Notification and Outcome of PND Module of the App

Regarding maternal PND, there were 4 grades of notifications sent to the mother—grade 0 (score lower than 25) was associated with a message indicating that everything is ok, grade 1 (score between 26 and 50) was associated with a recommendation to talk about symptoms with a close relative, grade 2 (score between 51 and 65) recommended that they quickly discuss their symptoms with a family doctor, and grade 3 (score higher than 65) recommended that they meet a family doctor as soon as possible. Grades 2 and 3 were considered a high probability for PND. This algorithm and questionnaire were the same as those used in our previous study [14].

To reduce PND incidence, we added, since 2022, a support program in the current version of the app with early and continuous advice after childbirth to lead parents to take care of their mental burden and to be aware of burnout and PND. We also provided them with the option of joining speaking groups and accessing testimonials from other mothers.

The last secondary outcome was the rate and the median time of the mothers’ PND notifications after childbirth and subsequently to the support and prevention program.

### Analysis

The analysis was performed on the 55,618 users, matching criteria were assessable for analysis and at least 1 neurodevelopment disorder strongly requiring a consultation was observed in 439 children.

Sensitivity, specificity, predictive positive and negative values, and the Youden index of algorithms triggering notifications of suspected NDDs were calculated according to the physician’s feedback. A notification was considered relevant if a physician suggested specific medical surveillance of the disorder or the consultation of an expert or a therapist or initiated therapy.

The *χ*^2^ test was used in 2×2 tables to assess the statistical association between the medical relevance of the notification (relevant or not) and the notification results (notification or no notification of a possible NDD). We also assessed the rate of probable PND of mothers having a score >50 in the survey and the changes in the rate of PND during the time after childbirth. The level of statistical significance was 5% for all statistical tests.

## Results

### Overview

**Figure 1:**
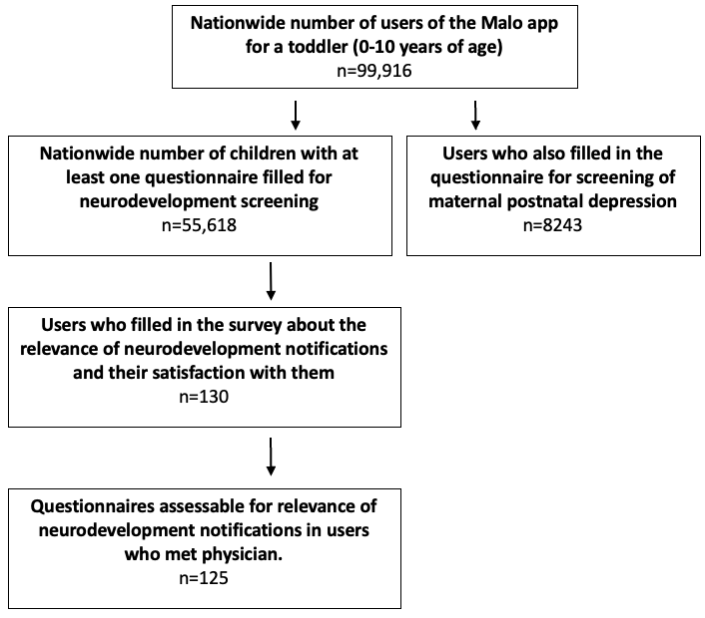
Flowchart of users of the Malo app.

**Figure 2:**
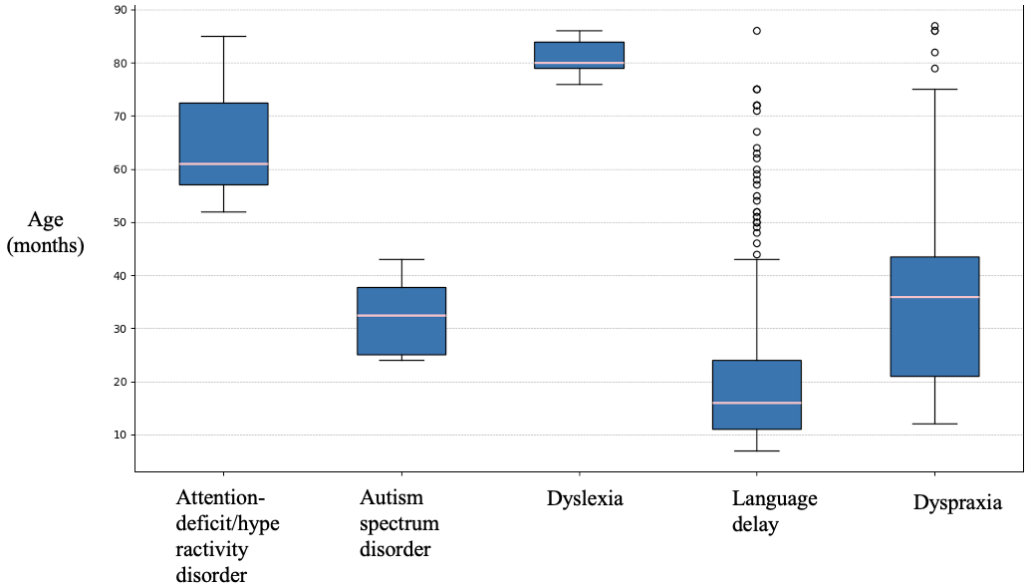
Distribution of the notifications of possible neurodevelopmental disorders and their type according to children’s age.

Among 99,916 nationwide users of the app between May 1, 2022, and February 8, 2024, a total of 55,618 children met the inclusion criteria (Figure 1). The median age of assessable children was 4 months (IQR 9), and 344,640 questionnaires were analyzed. Data analysis was performed at the end of February 2024. Among children, 0.8% (439/55,618) had at least 1 possible NDD strongly requiring a consultation. The median age of notification for probable ASD, language delay, dyspraxia, dyslexia, and ADHD was 32 (IQR 24-42), 16 (IQR 5-43), 36 (IQR 12-75), 80 (IQR 77-86) and 61 (IQR 52-85) months, respectively (Figure 2). The rate of probable ADHD, ASD, dyslexia, language delay, and dyspraxia in the population of children of the age included between the detection limits of each alert was 1.48%, 0.21%, 1.52%, 0.91%, and 0.37%, respectively.

### Analysis of the Assessment of the Relevance of the Alerts by Physicians

A 1-week survey was done at the end of January 2024 concerning the physician consultation feedback to parents following notification of possible NDD and to assess the satisfaction of users. Among the 130 parents who agreed to answer the survey, 113 had no alert, and 17 (13.1%) had received an alert of a possible NDD, which suggested a visit to their physician. Among users who received a notification suggesting a visit to their physician for a neurodevelopmental issue, 70.6% (12/17) users answered “YES” to the question “If you had a notification, did you follow the recommendation of the app to visit a physician?”

As 5 users with notification did not meet their physician, they were not included in the analysis of the clinical relevance of the alerts assessed by the physician. Data of the 125 assessable users who met the physician after notification showed a sensitivity of notification of 78.6%, a specificity of 98.2%, a positive predictive value of 84.6%, a negative predictive value of 97.3%, and a Youden index of 0.77 (*P*<.001).

Among the 11 children with true positive notifications of a possible NDD suggested by the app, medical surveillance of the evolution of the symptoms was proposed in 6 (54.5% of relevant notifications) cases, the advice of an expert was needed in 2 (18.2%) cases, and treatment was immediately initiated in 3 (27.3%) cases.

### Satisfaction Analysis

Among 130 users who filled in the satisfaction survey, 99.2% (129/130) found the app easy to use and 70% (91/130) reported that the app improved the follow-up of their child. Moreover, the app was rated 4.8/5 on Apple’s App Store with 990 votes and 4.7/5 on Android Stores with 1100 votes.

### Screening of PND

Among 8243 mothers who completed a PND questionnaire, highly probable PND (grades 2 or 3) was suspected in 938 (11.4%). Grades 1, 2, or 3 were reported in 54.2% (n=4468) of mothers. The a median 96 days (IQR 86) after childbirth and the incidence of supposed PND was equivalent during the 7-month follow-up (*P*<.001).

## Discussion

### Principal Findings

Our study prospectively assessed, in a “real-world” manner, in over 55,000 users, the benefit of mother-child dyad follow-up via a dedicated multidomain familial mHealth smartphone app providing early detection of 5 NDDs and leading to reduced incidence of maternal PND.

The main result is that the median age of alert for 5 NDDs (ASD, language delay, dyspraxia, dyslexia, and ADHD) by this smartphone screening app containing a dedicated questionnaire for parents seems to allow earlier assessment than in historical data. It also appears that the relevance of notifications was confirmed by physicians consulted following notification with high sensitivity (78.6%) and high specificity (98.2%).

Optimization of the neurodevelopment follow-up of children is very important as the identification of the first symptoms of NDDs is usually done by parents (without a dedicated digital device) in 61% of cases and by a health professional in only 14% as reported in a recent French study [7]. In our study, the median age of notification for probable ASD, ADHD, dyspraxia, dyslexia language delay was 32.5 (IQR 12.8), 61 (IQR 15.5), 36 (IQR 22.5), 80 (IQR 5), and 16 months (IQR 13), respectively, while time to diagnosis in France is usually 78 and 120 months for ASD and ADHD, respectively, that is, 4-5 years later, between 6 and 10 years of age for dyspraxia and dyslexia, and 2-3 years for language delay [7,8,15,17,18].

The incidence of each disorder suspected in our study was similar to those historically reported, strongly suggesting that our cohort was representative of the general population—0.21% for ASD in our study versus 0.6% in the literature [1], 1.48% for ADHD 0.4% versus 5% in the literature [19], and 0.91% for language delay versus 2% in France in the literature [20]. The rate of dyslexia was lower in our study (1.52%) than in the literature (3% to 6% [10,20]) as was the rate of dyspraxia: 0.37% in our study versus 3% in literature [20]. The lower rates of dyslexia and dyspraxia screening in our study are probably caused by a reduced sensibility of the algorithm.

We also performed an analysis of physician feedback after an alert about a possible NDD. Most users (13/18, 72.2%) followed the recommendation of the app to visit their family doctor or pediatrician after an alert. In our previous study published in 2022 with the older version of the NDD trigger algorithm, the rate of parents following the recommendation was 84.4% (54/64). Both results suggest a high level of confidence of parents in the notification [14].

The main modification of the algorithm and forms between the previous and current versions of the app consisted of reducing sensitivity to optimize specificity by displaying several sub-questionnaires over a more extended period (at least 1 month between early warnings and confirmation) for confirmation and investigation of suspicious symptoms on the main questionnaire. In this study, the sensibility of notification was reduced to 78.6% while it was 100% in the 2022 study, but the specificity was higher with new algorithms (98.2% vs 73.5%). This was a deliberate choice to improve specificity by reducing false positive alerts, as observed in the 2022 version of the app to reduce inopportune and anxiogenic alerts to parents. This is also associated with a high negative predictive value of 97.3% which is interesting to reassure parents in the absence of alerts. The high positive predictive value is also an important factor for the confidence of doctors confronted with an alert, delivered by the app. It suggested that 84.6% of notifications were considered relevant by a physician. Among these alerts, the physician triggered a specific medical surveillance in 54.5% of notifications initiated a treatment or recommended parents to an expert in 45.5%.

Although these data were declarative by users and were not directly confirmed by physicians, we assume that the specificity of the ASD notifications is close to the result of Pierce et al [6], showing overall stability or specificity of an autism spectrum diagnosis of 84% at earlier than 18 months of age through a universal screening program in primary care. In a recent diagnostic accuracy study including 13,511 children aged 11-42 months, Barbaro et al [21] showed 83% positive predictive value and 99% estimated negative predictive value of the Social Attention and Communication Surveillance-Revised tool for autism identification when it was used by nurses for children aged 12 months. Our results seem to be similar when parents perform a screening using our app.

The inclusion of efficient digital tools is important in the logic of care pathways because it promotes acceptability and relevance by families and professionals. Early screening allows for early diagnosis and interventions as reported by works on the efficacy of early treatments of cases among young children and recent promising studies on early interventions [9-11,22-25].

We also reported a lower incidence of maternal PND (11.4%, 938/8243) assessed in 8243 mothers than in our previous study performed in 2022 (16.6%, 157/907 assessed mothers). The form and algorithms were not modified for the part relating to maternal postpartum depression between the previous and current versions of the app. To prevent the PND rate, we added to the early PND screening a prevention program to be initiated by mothers after childbirth. PND is well known to disrupt the crucial mother-infant relationship on which optimal child development depends. It is the most common complication associated with childbirth, and it may exert harmful effects on children such as increased risk of ASD [26]. It is usually underdetected or detected after many months. The early treatment of PND is effective, avoids negative impact on child development, and does not necessarily require drugs to improve symptoms in the earliest stages [27]. Its prevalence in France is 18%.

In our previous study performed in 2022 with the old version of the app, we reported that 16.6% of mother users had probable PND and a median time of detection between 8 and 12 weeks after childbirth. We thus added a support program since 2022 in the new version of the app, consisting of advice, information, sensitization to PND, as well as access to speaking groups and testimonials of mothers. This study reported a lower rate of suspected PND of 11.4% (151/907), that is, a 31% reduction in PND incidence compared to the results of the 2022 study. There were no changes in the questionnaire; the same algorithm for PND detection was active in both versions of the app, but only the prevention program was added. Interestingly, the incidence of all grades of assessment (1, 2, or 3) was similar between both studies (56.7%, 515/907 mothers in the 2022 study vs 54.2%, 4465/8243), suggesting that a switch to lower grade symptoms was associated with the new support program which in turn was associated with a diminished incidence of PND. This is, as far as we know, the first time that a reduction in PND incidence can be observed through the use of a smartphone app.

The level of satisfaction was also high (between 70%, 91/130 and 99.2%, 129/130 according to the assessed domains) and contributed to the high rate of adoption.

### Limitations

There are limitations to our study. First, it was an observational study without a control group. Therefore, even though our sample was very significant, we could only proceed to an indirect (historical) comparison when intending to assess the efficacy of the tool regarding the detection of mental problems. Sample selection bias is always possible in the absence of randomization, due to social media recruitment modalities and because using the mobile app requires possession of a smartphone. We could have asked users questions about their educational level, practice classification (rural or urban), technical experience, and marital status, but we designed the app to collect as little personal data as possible. However, the very high rate of smartphone penetration in France (92% in a 2018 survey) in people aged 25-39 years led us to believe that the risk of a selection bias associated with smartphone use was low. Nonetheless, we do note that parents without smartphones cannot benefit from the app [28].

The second limitation is that the data were declarative by users without a comparative arm, but we found similar results to our previous study in terms of NDD incidence and time to the detection of benefit.

The third limitation is that NDD suspicions were not directly transmitted by physicians. As diagnostic confirmation takes time, prospective follow-up of patients can be interesting to assess if suspicion is confirmed and makes it possible to study the confirmation rate of suspicions.

Fourth, the attrition rate (ie, the discontinuation of eHealth app use) was not assessed, but it could be interesting to study whether the benefit of early detection of NDD is maintained over time, thanks to prolonged use [29]. We need further studies to improve the lack of follow-up rate which is usually high in real-life studies of eHealth instruments.

### Conclusions

To our knowledge, this multidomain mHealth app dedicated to both the early detection of 5 NDDs in children and the early detection and prevention of maternal PND is the first app with real-life data of clinical relevance. Results based on a large population of more than 55,000 users confirmed previous results and suggested that a multidomain familial mHealth app is suitable and effective for regular use in the mother-child dyad follow-up.

## Abbreviations

ADHD: attention-deficit/hyperactivity disorder
ASD: autism spectrum disorder
mHealth: mobile health
NDD: neurodevelopmental disorder
PND: postnatal depression

## Data Availability

The data sets generated and analyzed during this study are not publicly available due to the sensitive nature of the patient data and the risk for reidentification if crossed with other data sources but are available from the corresponding author on reasonable request.

## References

[1] GBD 2019 Mental Disorders Collaborators (2022). Global, regional, and national burden of 12 mental disorders in 204 countries and territories, 1990-2019: a systematic analysis for the Global Burden of Disease Study 2019. Lancet Psychiatry.

[2] Wetherby AM, Brosnan-Maddox S, Peace V, Newton L (2008). Validation of the Infant-Toddler Checklist as a broadband screener for autism spectrum disorders from 9 to 24 months of age. Autism.

[3] Turner-Brown LM, Baranek GT, Reznick JS, Watson LR, Crais ER (2013). The First Year Inventory: a longitudinal follow-up of 12-month-old to 3-year-old children. Autism.

[4] Chlebowski C, Robins DL, Barton ML, Fein D (2013). Large-scale use of the modified checklist for autism in low-risk toddlers. Pediatrics.

[5] Robins DL, Casagrande K, Barton M, Chen CMA, Dumont-Mathieu T, Fein D (2014). Validation of the modified checklist for autism in toddlers, revised with follow-up (M-CHAT-R/F). Pediatrics.

[6] Pierce K, Gazestani VH, Bacon E, Barnes CC, Cha D, Nalabolu S, Lopez L, Moore A, Pence-Stophaeros S, Courchesne E (2019). Evaluation of the diagnostic stability of the early autism spectrum disorder phenotype in the general population starting at 12 months. JAMA Pediatr.

[7] Ce Que Nous Disent les Personnes et les Familles sur Leur Parcours de Vie. Ipsos.

[8] Attention deficit disorder with or without hyperactivity: identifying suffering, supporting the child and the family - questions/answers. Haute Autorité de Santé.

[9] Wallace IF, Berkman ND, Watson LR, Coyne-Beasley T, Wood C, Cullen K, Lohr KN (2015). Screening for speech and language delay in children 5 years old and younger: a systematic review. Pediatrics.

[10] Wagner RK, Zirps FA, Edwards AA, Wood SG, Joyner RE, Becker BJ, Liu G, Beal B (2020). The prevalence of dyslexia: a new approach to its estimation. J Learn Disabil.

[11] Tamplain P, Miller HL, Peavy D, Cermak S, Williams J, Licari M (2024). The impact for DCD - USA study: the current state of developmental coordination disorder (DCD) in the United States of America. Res Dev Disabil.

[12] Hahn-Holbrook J, Cornwell-Hinrichs T, Anaya I (2018). Economic and health predictors of national postpartum depression prevalence: a systematic review, meta-analysis, and meta-regression of 291 studies from 56 countries. Front Psychiatry.

[13] O'Connor E, Rossom RC, Henninger M, Groom HC, Burda BU (2016). Primary care screening for and treatment of depression in pregnant and postpartum women: evidence report and systematic review for the US preventive services task force. JAMA.

[14] Denis F, Maurier L, Carillo K, Ologeanu-Taddei R, Septans AL, Gepner A, Le Goff F, Desbois M, Demurger B, Silber D, Zeitoun J, Assuied GP, Bonnot O (2022). Early detection of neurodevelopmental disorders of toddlers and postnatal depression by mobile health app: observational cross-sectional study. JMIR Mhealth Uhealth.

[15] Neurodevelopmental disorders identification and guidance of children at risk. Summary. Haute Authorité de Santé.

[16] Jullien S (2021). Screening for autistic spectrum disorder in early childhood. BMC Pediatr.

[17] Habib M (2021). The neurological basis of developmental dyslexia and related disorders: a reappraisal of the temporal hypothesis, twenty years on. Brain Sci.

[18] Bazen L, van den Boer M, de Jong PF, de Bree EH (2020). Early and late diagnosed dyslexia in secondary school: performance on literacy skills and cognitive correlates. Dyslexia.

[19] Polanczyk GV, Willcutt EG, Salum GA, Kieling C, Rohde LA (2014). ADHD prevalence estimates across three decades: an updated systematic review and meta-regression analysis. Int J Epidemiol.

[20] DYS disorders. Calameo.

[21] Barbaro J, Sadka N, Gilbert M, Beattie E, Li X, Ridgway L, Lawson LP, Dissanayake C (2022). Diagnostic accuracy of the social attention and communication surveillance-revised with preschool tool for early autism detection in very young children. JAMA Netw Open.

[22] Ha S, Han JH, Ahn J, Lee K, Heo J, Choi Y, Park JY, Cheon KA (2022). Pilot study of a mobile application-based intervention to induce changes in neural activity in the frontal region and behaviors in children with attention deficit hyperactivity disorder and/or intellectual disability. J Psychiatr Res.

[23] Siu AL, Bibbins-Domingo K, Grossman DC, Baumann LC, Davidson KW, Ebell M, García FAR, Gillman M, Herzstein J, Kemper AR, Krist AH, Kurth AE, Owens DK, Phillips WR, Phipps MG, Pignone MP, US Preventive Services Task Force (USPSTF) (2016). Screening for autism spectrum disorder in young children: US preventive services task force recommendation statement. JAMA.

[24] Landa RJ (2018). Efficacy of early interventions for infants and young children with, and at risk for, autism spectrum disorders. Int Rev Psychiatry.

[25] Stahmer AC, Dababnah S, Rieth SR (2019). Considerations in implementing evidence-based early autism spectrum disorder interventions in community settings. Pediatr Med.

[26] Pham C, Symeonides C, O'Hely M, Sly PD, Knibbs LD, Thomson S, Vuillermin P, Saffery R, Ponsonby AL, Barwon Infant Study Investigator Group (2022). Early life environmental factors associated with autism spectrum disorder symptoms in children at age 2 years: a birth cohort study. Autism.

[27] Holt C, Gentilleau C, Gemmill AW, Milgrom J (2021). Improving the mother-infant relationship following postnatal depression: a randomised controlled trial of a brief intervention (HUGS). Arch Womens Ment Health.

[28] Digital Barometer 2018. Secrétariat d’État au numérique.

[29] Eysenbach G (2005). The law of attrition. J Med Internet Res.

